# Veterinary World reviewer acknowledgment 2019

**DOI:** 10.14202/vetworld.2020.538-541

**Published:** 2020-03-23

**Authors:** A. V. Sherasiya, Nazir

**Affiliations:** Veterinary World, Star, Gulshan Park, NH-8A, Chandrapur Road, Wankaner – 363621, Dist. Morbi, Gujarat, India

**Contributing reviewers**

Veterinary World editorial team would sincerely like to thank all of our reviewers who contributed to peer review for the journal in 2019.

**A. A. Swelum** Saudi Arabia

**A. Dusty Miller** USA

**A. I. Gelasakis** Greece

**A. K. Bulashev** Kazakhstan

**Abbassi Mohamed Salah** Tunisia

**Abdelkbir Rhalem** Morocco

**Abdullah Karasu** Turkey

**Abid Hussain Shahzad** Pakistan

**Ademola Adetokunbo Oyagbemi** Nigeria

**Adeyinka Jeremy Adedeji** Nigeria

**Adriana Titilincu** Romania

**Ahmad Daryani** Iran

**Ahmed Ali Mahmoud Saleh** Japan

**Ahmed S Abdoon** Egypt

**Ailian Geng** China

**Alejandro Hidalgo** Colombia

**Alfredo Medrano** Mexico

**Ali Abbas Nikvand** Iran

**Ali Yousefi** Iran

**Amitava Dey** India

**Amlan K. Patra** India

**Ammar Ahmad Khan** Pakistan

**Anja Joachim** Austria

**Anthony Flores** USA

**Antoni Prenafeta** Spain

**Antonio Bevilacqua** Italy

**Anwar M Hashem** Saudi Arabia

**Arda Sozcu** Turkey

**Arda Yildirim** Turkey

**Arya Sobhakumari** USA

**Ashish Chopra** India

**Ashwani Kumar** India

**Ayman Abdel-Aziz Swelum** Egypt

**B. D. Enger** USA

**Banan Khalid Al-Baggou** Iraq

**Baratang Alison Lubisi** South Africa

**Barbara Kot** Poland

**Belgin Siriken** Turkey

**Bersissa Kumsa, Eseta** Ethiopia

**Bhoj Raj Singh** India

**Breda Jakovac-Strajn** Slovenia

**Breno Valentim Nogueira** Brazil

**Buket Er Demirhan** Turkey

**C. M. Franco** Spain

**Chandramani Pathak** India

**Chao-Nan Lin** Taiwan

**Charleata A Carter** USA

**Christopher Didigwu Nwani** Nigeria

**Claude T Sabeta** South Africa

**Claudia Niemeyer-Svedberg** Brazil

**Daniel M. Aguiar** Brazil

**Danielle Bastos Araujo** Brazil

**David Carmena** Spain

**David Gleeson** Ireland

**Deepak Barot** India

**Denisa Soledad Pérez Gaudio** Argentina

**Domingo Fernández García** Spain

**Ashok Dangi** India

**Sreenu Makkena** India

**Archana Jain** India

**Kalyan Sundar Das** India

**Riaz Hussain** Pakistan

**Pourouchottamane R.** India

**Dragica Salamon** Croatia

**Edward Ferguson** USA

**Ehsan Mostafavi** Iran

**Ekene Vivienne Ezenduka** Nigeria

**Elangovan AV** India

**Elina B. Reinoso** Argentina

**Emiliano Lasagna** Italy

**Felipe da Silva Krawczak** Brazil

**Filippo Fratini Fratini** Italy

**Flaviu Alexandru Tabaran** Romania

**Florence David Tarfa** Nigeria

**Fufa Ido Gimba** Malaysia

**G. Greif** Uruguay

**G. Schares** Germany

**Gael D. Maganga** Gabon

**Gamal Wareth** Germany

**Genevieve Andre-Fontaine** France

**Gnanavel Venkatesan** India

**Gregório Guilherme Almeida** Brazil

**Gul Ahmad** USA

**Guo-zhong Zhang** China

**Hamed Kalani** Iran

**Hamid Kassiri** Iran

**Hany Mhany Ragab Abdel-Latif** Egypt

**Harvie P. Portugaliza** Philippines

**Hassan A. Hussein Hussein** Egypt

**Hazem Shaheen** Egypt

**Hehsam N. Mustafa** Saudi Arabia

**Hind Yahyaoui Azami** Switzerland

**Hongning Wang** China

**Hua-Ji Qiu** China

**Hudson A. Pinto** Brazil

**Hui-Chen Guo** China

**Huiling Sun** China

**Huseyin Yilmaz** Turkey

**I. Giannenas** Greece

**Iddya Karunasagar** India

**Indrasen Chauhan** India

**Inga Stadaliene** Lithuania

**Ingrid Bortolin Affonso Lux Hoppe** Brazil

**Ismail Hakkı Tekiner** Turkey

**J. Hafid** Morocco

**Jamal Gharekhani** Iran

**Jean-Mathieu Bart** France

**Jemma Bergfeld** Australia

**Jerome Andonissamy** India

**Jie cheng** China

**Jose Edmundo Apraez Guerrero** Colombia

**Josef illek** Czech Republic

**Joseph Bosilevac** USA

**Joshua Mbanga** Zimbabwe

**Juan Morgaz** Spain

**Kannan Thandavan Arthanari** India

**Kavous Solhjoo** Iran

**Kekungu Puro** India

**Keshan Zhang** China

**Kiril M. Dimitrov** USA

**Kosta Y. Mumcuoglu** Israel

**Koushlesh Ranjan** India

**Laxmi Narayan Sarangi** India

**Leonardo Alves Rusak** Brazil

**Liben Chen** USA

**Linous Munsimbwe** Zambia

**Magdalena Zając** Poland

**Mahmoud Mohey Elhaig** Egypt

**Maja Josip Velhner** Serbia

**María de Lourdes Adrien Delgado** Uruguay

**Maria Emilia Eirin** Argentina

**Maria Miguel** Portugal

**Mariette Ducatez** France

**Martina Floeck** Austria

**Mirosław Mariusz Michalski** Poland

**Mochamad Syaifudin** Indonesia

**Mohammad Mijanur Rahman** Malaysia

**Mohammed El-Houadfi** Morocco

**Mohammed Hamdy Farouk** China

**Moutaz Zarkawi** Syrian Arab Republic

**Mustafa Cengiz** Turkey

**Myriam Oudni-M’rad** Tunisia

**N. D. Sargison** UK

**Naif Khalaf Alharbi** Saudi Arabia

**Naveen Kondru** USA

**Nazli Ercan** Turkey

**Neha Gogia** USA

**Nuriye Ezgi Bektur** Turkey

**Olfat Anter Mahdy** Egypt

**P. L. Toutain** France

**Padet Siriyasatien** Thailand

**Paul M. Bartley** UK

**Pınar Şanlıbaba** Turkey

**Piyumali Kanchana Perera** Australia

**Prabhat Kumar Pankaj** India

**Pradeep Kumar Malik** India

**Prem Singh Yadav** India

**Pwaveno Huladeino Bamaiyi** Malaysia

**R. Umaya Suganthi** India

**Rafael Cardoso Carvalho** Brazil

**Rajani Ravindranath Thanissery** USA

**Rajender Kumar** India

**Rajib Deb** India

**Ramute Miseikiene** Lithuania

**Raquel Salazar Lugo** Venezuela

**Richard Churchchil** India

**Roberto Condoleo** Italy

**Roman R. Ganta** USA

**Rute M. Noiva** Portugal

**S. B. N. Rao** India

**S. Mathan Kumar** Oman

**S. Liu** China

**Sabry A. El-khodery** Egypt

**Saganuwan Alhaji Saganuwan** Nigeria

**Sandrine Musa** Germany

**Satoshi Sekiguchi** Japan

**Serena Montagnaro** Italy

**Shamim Sarkar** Bangladesh

**Shannon K. French** Canada

**Shawky M. Aboelhadid** Egypt

**Shoja Jafari** Iran

**Sinan Ince** Turkey

**Sofía Grecco** Uruguay

**Sorin Daniel Dan** Romania

**Sprygin Alexander** Russia

**Sreekumar Chirukandoth** India

**Stuart Gordon** New Zealand

**Sunil More** USA

**Supaluk Popruk** Thailand

**Tanko Polycarp Nwunuji** Nigeria

**Tapas Kumar Sar** India

**Teodulo Quezada Tristan** Mexico

**Thomas J Nolan** USA

**Tomas Erban** Czech Republic

**Umberto Molini** Namibia

**V. C. Rayulu** India

**Vahid Noaman** Iran

**Valter Luiz Maciel** Jr. Brazil

**Varij Nayan** India

**Vijaya Kumar Anumolu** India

**Vikas Vohra** India

**Vishal S. Suthar** India

**Walaa Fathy Saad** Egypt

**Walied Abdo** Egypt

**Wenxiao Jiang** China

**Widya Paramita Lokapirnasari** Indonesia

**Wojciech Zygner** Poland

**Xu Gu** China

**Ya-Ching Lin** Taiwan

**Ya-Ling Huang** Taiwan

**Yasmina Mohammed Abd El Hakim El Ghazaly** Egypt

**Yasser M. Albadrany** Iraq

**Yongning Zhang** China

**Zahid Naseer** Pakistan

**Zahra Boroomand** Iran

**Zhuo-Ming Qin** China

**Zuhair Ismail** Jordan

